# Analysis of Clinical and Epidemiological Profiles as Predictors of Complications in Women Admitted to the Acute Cardiac Care Unit for Acute Coronary Syndrome

**DOI:** 10.1089/whr.2025.0005

**Published:** 2025-05-08

**Authors:** Marta Parellada-Vendrell, Sílvia Pérez-Ortega, Nuria Romeu-Mirabete, Montserrat Prat-Masana, Montserrat Venturas, Adelaida Zabalegui, Rut Andrea

**Affiliations:** ^1^ Acute Cardiac Care Unit, Cardiovascular Clinical Institute, Hospital Clínic of Barcelona, Barcelona, Spain.; ^2^ University of Barcelona, Barcelona, Spain.; ^3^ Cardiovascular Clinical Institute , Hospital Clínic of Barcelona, Barcelona, Spain.; ^4^ IDIBAPS, August Pi i Sunyer Biomedical Research Institute, Hospital Clínic of Barcelona, Barcelona, Spain.; ^5^ Subdivision of Research and Teaching in Nursing, Hospital Clínic of Barcelona, Barcelona, Spain.

**Keywords:** acute coronary syndrome (ACS), critical care nursing, women’s health, heart disease risk factors (CVRFs), myocardial infarction with non-obstructive coronary arteries (MINOCA), gender and health

## Abstract

**Introduction:**

In women, cardiovascular disease accounts for 35% of annual deaths, with ischemic heart disease being the leading cause. There are knowledge gaps in research, prevention, treatment, and access to cardiovascular care in women.

**Objectives:**

To describe the clinical and epidemiological profiles of women with acute coronary syndrome (ACS) admitted to an Acute Cardiac Care Unit (ACCU) and to study their association with the development of complications.

**Methods:**

This descriptive study included women admitted to the ACCU of a tertiary hospital for ACS. Sociodemographic, anthropometric, and clinical variables were assessed. Descriptive and inferential statistical analyses were performed using the SPSS v25 software.

**Results:**

Eighty women (mean age, 68 ± 13 years) with a high prevalence of cardiovascular risk factors were included, and 66.2% presented ST-segment elevation ACS. The prevailing symptom was chest pain in 96.3% of patients, followed by associated symptoms such as sweating, nausea, and dyspnea (86.3%). The etiology was secondary to obstructive coronary artery disease in 81.3%, and the therapeutic strategy was percutaneous coronary intervention in 72.5% of patients. Among the women, 64.1% attributed their symptoms to non-cardiac causes, 60% did not perceive severity, and 35.2% presented complications of ACS, particularly cardiac arrest and arrhythmias. A reduced ejection fraction and being alone at the onset of symptoms were associated with a higher risk of complications.

**Conclusions:**

Despite severe clinical presentations and complications, women have a low perception of severity and attribute cardiovascular symptoms to non-cardiac causes. Increasing awareness of ACS and its complications in women is needed among the population in order to improve health outcomes.

## Introduction

Cardiovascular disease (CVD) is the leading cause of death worldwide. According to the *Global Burden of Cardiovascular Diseases and Risk Factors*, which evaluated the global burden of CVDs and their risk factors using data from 1990 to 2019, the prevalence, mortality, and disability-adjusted life years and years of life lost increased significantly during this period.^1^ The two entities that caused the highest mortality and disability were ischemic cardiomyopathy and stroke. Certain modifiable risk factors (cardiometabolic, behavioral, environmental, and social) can be addressed to improve the cardiovascular health of the population, thus reducing the risk of premature death and health care costs.^1^

Historically, epidemiological studies, mainly those centered on coronary disease, were focused on the male population since ischemic cardiomyopathy was believed to be a health condition that mainly affected men, and thus, women were excluded from clinical trials.^2^ In this same sense, *Healy B* published the “Yentl Syndrome” in the *New England Journal of Medicine* in 1991, where she refers to the medical invisibility of women in studies on CVD and where she exposed that women had to behave according to male clinical canons in order to receive the same health care or they were underdiagnosed and undertreated otherwise, causing a decrease in the quality and effectiveness of care.^3^

Knowledge gaps in research, prevention, treatment, and access to care have endured until now, despite CVDs in women being responsible for 35% of annual deaths and ischemic cardiomyopathy being the leading cause of mortality in this population group. Even the models used in risk assessment do not consider women-specific risk factors, gender perspectives, or other determining factors such as psychosocial or socioeconomic factors.^4^

Advances in cardiology, as well as the strategy of scientific societies with the implementation and updating of clinical practice guidelines on acute coronary syndrome (ACS), are aimed at early detection, optimizing the management, and treatment of these patients, and the disparity between sexes is still present and a challenge for the future.^5^ For years, different national and international strategies have been implemented to reduce ischemic time in ACS, such as reference centers with infarction codes and access to 24/7 cardiac catheterization, activation of the infarction code from the out-of-hospital medical emergency service, the European *Stent for Life* initiative, and chest pain care units in the emergency department. The purpose of these initiatives is for anyone diagnosed with ACS to have early and equal access to the coronary reperfusion treatment.

*The Lancet Women and Cardiovascular Disease Commission*^4^ is aligned with the United Nations’ Sustainable Development Goals and aims to reduce the global burden of CVD in women by 2030 by educating health care providers and patients on the early detection of CVD, expanding access to cardiovascular health programs, and prioritizing research and intervention strategies for CVD in women.

Based on these premises, the main objective of this study was to analyze the clinical and epidemiological profiles of women with ACS admitted to an Acute Cardiac Care Unit (ACCU), compare ACS groups, and emphasize social and anthropometric factors, perception of the disease, and access to diagnostic and therapeutic procedures, which are associated with the development of complications.

## Materials and Methods

### Study design

This was a descriptive study of all women admitted to the ACCU of a high-tech public university hospital for ACS between January 2022 and January 2023.

### Study population

The study population included all women diagnosed with ACS who were admitted to the ACCU, referred from other hospitals, or from the hospital itself, consecutively selected by non-probabilistic convenience sampling, and who signed the informed consent form for the study. Women with cognitive impairment, confusional syndrome, or language barriers and those who were admitted to the unit for a cardiac pathology other than ACS were excluded.

### Scope of the study

Our center is a highly complex tertiary hospital that had an ACCU with 16 beds during the inclusion period of the study, 8 for critical care and 8 for intermediate care, 1 of which is specifically dedicated to the care of the infarction code.

### Sample size

The sample size included all women who were consecutively admitted for ACS to the ACCU during the study period and met all the inclusion criteria and none of the exclusion criteria.

### Collection of variables

A data collection sheet was designed *ad hoc* before starting the recruitment period, and a meeting was held with the research team to clarify concepts and avoid biases in the research, as well as to carry out a pilot test.

The study variables were divided into the following categories:

• Sociodemographic data included age, marital status, occupation, place of residence, and dependent persons.
• Anthropometric data: weight, height, body mass index (BMI), and classification according to BMI.
• Cardiovascular risk factors (CVRFs): post-menopause, dyslipidemia, hypertension, family history of ischemic cardiomyopathy, perceived stress, mixed anxiety-depressive disorder, diabetes mellitus, active tobacco use, ex-tobacco use, chronic renal failure, history of ischemic cardiomyopathy, alcohol abuse, and substance abuse.
• Clinical manifestations during the acute event include the presence of chest pain, intensity, thoracic and non-thoracic location (epigastric, mandibular, arms, or back), pain characteristics, radiation, and associated symptoms.
• Diagnosis and therapy: electrocardiographic classification of ACS, type of ACS, Killip–Kimball classification, priority of coronarography, ACS etiology, therapeutic strategy, complications arising from ACS, and left ventricular ejection fraction (LVEF).

Data collection did not modify the visits or activities typical of clinical practice. Clinical data were prospectively collected through a review of hospital medical records and direct interviews with the participants. The interview was conducted 12 hours after admission to the ACCU, lasting approximately 45–60 minutes, provided that the participant was conscious, oriented, hemodynamically stable, and agreed to participate in the study.

Cases that did not meet these criteria, either due to hemodynamic instability or the impossibility of verbal communication due to orotracheal intubation or sedation, were re-evaluated on a daily basis until the aforementioned conditions were met or they were considered as losses.

### Statistical analysis

The data collected were subjected to descriptive and inferential statistical analyses and were compared based on the electrocardiographic classification of ACS, forming two analysis groups: women with ST-segment elevation acute coronary syndrome (STEACS) and women with non-ST-segment elevation acute coronary syndrome (NSTEACS).

The Kolmogorov–Smirnov test was used to determine the normal distribution of continuous variables. In the descriptive analysis, categorical variables were expressed as absolute frequencies and percentages, whereas quantitative variables with a normal distribution were expressed as the mean with standard deviation or, alternatively, as the median and interquartile range.

In the inferential analysis, the Student’s *t-*test or Mann–Whitney U test was applied for quantitative variables, the chi-square test and Fisher’s exact test for qualitative variables, and the Kruskal–Wallis test for ordinal variables. A bilateral value of *p* < 0.05 was considered significant. The relative risk was calculated using a 95% confidence interval as an epidemiological measure of association. Statistical analyses were performed using IBM SPSS Statistics v25.

### Ethical considerations

The study complied with the standards of the Declaration of Helsinki and was approved by the Drug Research Ethics Committee of our center (registration code: HCB/2021/1221).

All the participants received written and verbal information regarding the study and signed an informed consent form.

## Results

**FIG. 1.**
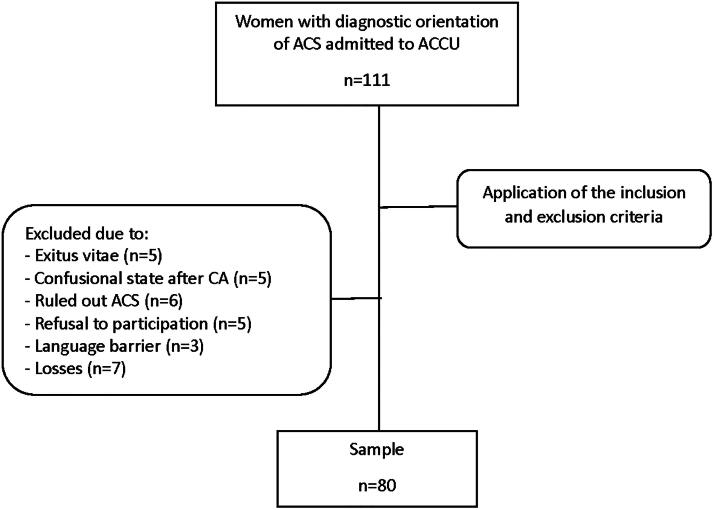
Flowchart. ACCU, Acute Cardiac Care Unit; ACS, acute coronary syndrome; CA, cardiorespiratory arrest.

During the study period, 111 women were admitted to the ACCU with diagnostic orientation for ACS. After applying the inclusion and exclusion criteria, 31 women were excluded for various reasons, as shown in the flowchart (Fig. 1).

### Characteristics of the population and ACS

The final sample comprised 80 participants, with a mean age of 68 ± 13 years. The age distribution showed an increasing trend of ACS from 51 years onwards, with the age group 71–80 years having the highest prevalence of 30%. Regarding marital status, occupation, and place of residence, 50% of the women were married, 23.8% were widowed, 61.3% were pensioners, and 75% lived in Barcelona City, with no statistically significant differences between the two ACS groups. A total of 23.8% had dependent persons, emphasizing the role of women as caregivers for minor children (31.6%), grandchildren (26.3%), dependent relatives (15.8%), and children with physical or mental health problems (15.8%) (Table 1).

**Table 1.** Sociodemographic Characteristics Distributed According to ACS

Variables	Total *N* = 80 (%)	STEACS *N* = 53 (%)	NSTEACS *N* = 27 (%)	*p*-Value
Age groups				
Age (mean ± SD)	68 ± 13	68 ± 13	67 ± 12	0.788
≤40 years	2 (2.5)	1 (1.9)	1 (3.7)	0.754
41–50 years old	5 (6.3)	4 (7.5)	1 (3.7)	
51–60 years old	15 (18.8)	10 (18.9)	5 (18.5)	
61–70 years old	19 (23.8)	12 (22.6)	7 (25.9)	
71–80 years old	24 (30)	14 (26.4)	10 (37)	
>80 years	15 (18.8)	12 (22.6)	3 (11.1)	
Marital status				
Single	7 (8.8)	2 (3.8)	5 (18.5)	0.140
Cohabitation	2 (2.5)	2 (3.8)	0 (0)	
Married	40 (50)	27 (50.9)	13 (48.1)	
Separated—divorced	12 (15)	7 (13.2)	5 (18.5)	
Widowed	19 (23.8)	15 (28.3)	4 (14.8)	
Occupation				
Housekeeping	10 (12.5)	6 (11.3)	4 (14.8)	0.276
Working	21 (26.3)	17 (32.1)	4 (14.8)	
Pensioner	49 (61.3)	30 (56.6)	19 (70.4)	
Dependent persons				
Yes	19 (23.8)	15 (28.3)	4 (14.8)	0.267
Minor children (*n* = 19)	6 (31.6)	5 (33.3)	1 (25)	0.516
Grandchildren	5 (26.3)	3 (20)	2 (50)	
Dependent family member (*n* = 19)	3 (15.8)	3 (20)	0 (0)	
Child with mental or physical health issues (*n* = 19)	3 (15.8)	3 (20)	0 (0)	
Husband and/or adult child (*n* = 19)	2 (10.5)	1 (6.7)	1 (25)	
Place of residence				
Barcelona (city)	60 (75)	38 (71.7)	22 (81.5)	0.294
Other towns in greater Barcelona	17 (21.3)	13 (24.5)	4 (14.8)	
Another province	1 (1.3)	0 (0)	1 (3.7)	
Other country	2 (2.5)	2 (3.8)	0 (0)	

The results are expressed as mean ± standard deviation or *n* (%).

ACS, acute coronary syndrome; NSTEACS, non-ST-segment elevation acute coronary syndrome; SD, standard deviation; STEACS, ST-segment elevation acute coronary syndrome.

**FIG. 2.**
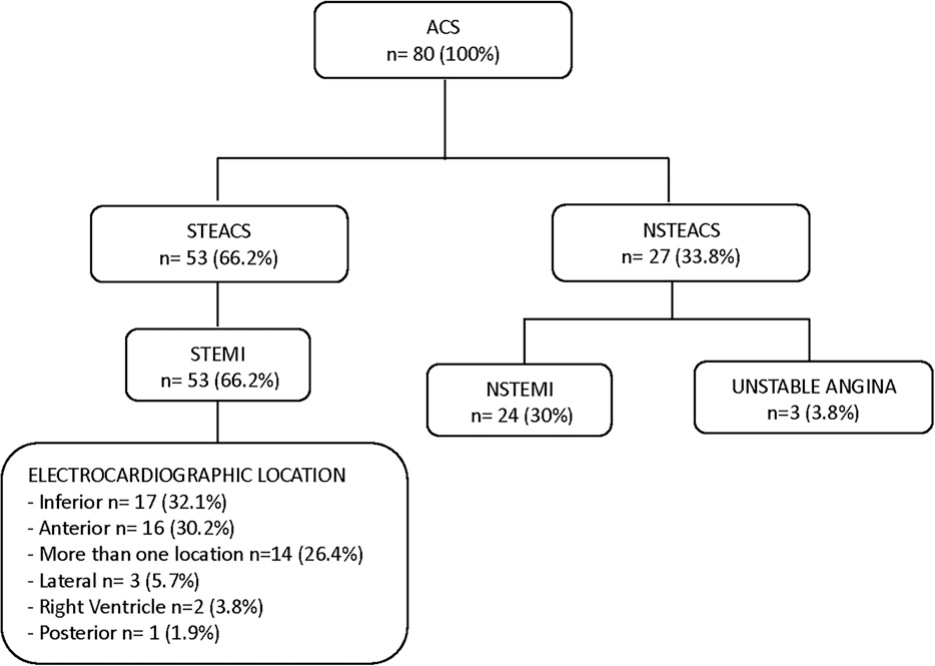
ACS classification and location of STEMI. The results are expressed as *n* (%). ACS, acute coronary syndrome; NSTEACS, non-ST-segment elevation acute coronary syndrome; NSTEMI, non-ST-elevation myocardial infarction; STEACS, ST-segment elevation acute coronary syndrome; STEMI, ST-elevation myocardial infarction.

According to the electrocardiogram recorded during the acute episode, 66.2% had a STEACS that was diagnosed as ST-elevation myocardial infarction (STEMI) versus 33.8% who showed an NSTEACS and a pathological correlation of non-ST-elevation myocardial infarction (NSTEMI) in 30% and unstable angina in 3.8% (Fig. 2). Figure 2 also shows the classification of STEMI according to electrocardiographic location, where the predominant lesions occurred on the inferior and anterior sides.

Of the sample, 52.5% had a high CVRF burden, defined as the coexistence of five or more factors, including post-menopausal status in 87.3%, dyslipidemia in 63.7%, hypertension in 61.3%, family history of ischemic heart disease in 41.3%, and perceived stress in 36.3%. From an anthropometric point of view, although no statistical significance was found, it should be noted that 37.5% of the patients were overweight (BMI 25–29.9 kg/m^2^), and 28.8% were obese (BMI ≥30 kg/m^2^). In addition, a statistically significant association was found between active tobacco use and STEACS (39.6% vs. 11.1%; *p* = 0.010) and between a personal history of ischemic heart disease and NSTEACS (9.4% vs. 29.6%; *p* = 0.021) (Table 2).

**Table 2.** Distribution of Anthropometric Features and Risk Factors According to ACS

Variables	Total *N* = 80 (%)	STEACS *N* = 53 (%)	NSTEACS *N* = 27 (%)	*p*-Value
Weight, median (IQR)	65.5 (80–60.2)	68 (80–61)	65 (75–58)	0.280
Height, median (IQR)	160 (165–152.2)	160 (164.5–152.5)	160 (165–150)	0.874
BMI, median (IQR)	27 (31.2–23.8)	27.1 (31.8–24.1)	26 (29.8–22.2)	0.306
Classification according to BMI				
Underweight (BMI <18.5)	3 (3.8)	1 (1.9)	2 (7.4)	0.331
Regular weight (BMI 18.5–24.9)	24 (30)	16 (30.2)	8 (29.6)	
Overweight (BMI 25–29.9)	30 (37.5)	18 (34)	12 (44.4)	
Obesity (BMI ≥30)	23 (28.7)	18 (34)	5 (18.5)	
Cardiovascular risk factors				
Number of CVRFs, median (IQR)	5 (6–3)	5 (6–3.5)	5 (6–3)	0.804
Coexistence of CVRFs				
<5 CVRFs	38 (47.5)	26 (49.1)	12 (44.4)	0.696
≥5 CVRFs	42 (52.5)	27 (50.9)	15 (55.6)	
Post-menopause	69 (87.3)	44 (83)	25 (96.2)	0.153
DLP	51 (63.7)	31 (58.5)	20 (74.1)	0.170
HBP	49 (61.3)	30 (56.6)	19 (70.4)	0.232
Family history of ischemic cardiomyopathy	33 (41.3)	23 (43.4)	10 (37)	0.585
Perceived stress	29 (36.3)	31 (39.6)	8 (29.6)	0.379
Mixed anxiety-depressive disorder	28 (35)	20 (37.7)	8 (29.6)	0.472
DM	25 (31.3)	14 (26.4)	11 (40.7)	0.191
Type 1 (*n* = 25)	2 (8)	1 (7.1)	1 (9.1)	1.000
Type 2 (*n* = 25)	23 (92)	13 (92.9)	10 (90.9)	1.000
Active tobacco use	24 (30)	21 (39.6)	3 (11.1)	**0.010**
Ex-tobacco use	15 (18.8)	8 (15.1)	7 (25.9)	0.241
Chronic renal failure	15 (18.8)	9 (17)	6 (22.2)	0.570
History of ischemic cardiomyopathy	13 (16.3)	5 (9.4)	8 (29.6)	**0.021**
Alcohol abuse	2 (2.5)	2 (3.8)	0 (0)	0.547
Substance abuse	1 (1.3)	1 (1.9)	0 (0)	1.000

Bold values represent *p* < 0.05 statistically significant.

The results are expressed as median (interquartile range) or *n* (%).

BMI, body mass index; CVRF, cardiovascular risk factors; DLP, dyslipidemia; DM, diabetes mellitus; HBP, high blood pressure; IQR, interquartile range.

Regarding clinical presentation and according to the definition of chest pain established by the *American Heart Association’s Guideline for the Evaluation and Diagnosis of Chest Pain*,^6^ 96.3% reported chest pain, 73.7% central thoracic pain, 82.7% with oppressive characteristics, 74% radiated pain, and 74% severe intensity according to the verbal numerical rating scale, with no differences between women with STEACS and NSTEACS. A total of 86.3% of patients presented with other associated clinical manifestations, emphasizing the presence of more than four symptoms in 21.7%. The reported symptoms were, in order of prevalence, 56.5% sweating, with a statistical association between this and STEACS (66% vs. 36.4%; *p* = 0.021), 40.6% nausea, 36.2% dyspnea, and 27.5% weakness, among other manifestations (Table 3). When inquiring about the possible origin of the symptoms and their perception of severity, 64.1% of the women attributed the symptoms to non-cardiac causes (STEACS 60.8% vs. NSTEACS 70.4%; *p* = 0.401), and 60% did not perceive the severity of the situation (STEACS 56.6% vs. NSTEACS 66.7%; *p* = 0.385).

**Table 3.** Clinical Manifestations During the Acute Event, Distributed According to ACS

Variables	Total *N* = 80 (%)	STEACS *N* = 53 (%)	NSTEACS *N* = 27 (%)	*p*-Value
Presence of thoracic pain	77 (96.3)	51 (96.2)	26 (96.3)	0.988
Pain intensity: VNRS (0–10)				
Median VNRS (IQR)	8 (10–7)	8 (10–8)	8 (9–7.5)	0.204
Mild (VNRS <3)	0 (0)			0.405
Moderate (VNRS 4–7)	19 (26)	11 (22.9)	8 (32)	
Severe (VNRS >8)	54 (74)	37 (77.1)	17 (68)	
Pain location				
Central thoracic	56 (73.7)	36 (70.6)	20 (80)	0.700
Precordial	9 (11.8)	6 (11.8)	3 (12)	
Epigastric	5 (6.6)	4 (7.8)	1 (4)	
Back of the chest	3 (3.9)	3 (5.9)	0 (0)	
Mandibular	1 (1.3)	1 (2)	0 (0)	
Left arm	1 (1.3)	1 (2)	0 (0)	
Right hemithorax	1 (1.3)	0 (0)	1 (4)	
Pain traits				
Oppressive	62 (82.7)	41 (82)	21 (84)	0.444
Burning	7 (9.3)	5 (10)	2 (8)	
Sharp	3 (4)	1 (2)	2 (8)	
Ripping	3 (4)	3 (6)	0 (0)	
Presence of irradiation	57 (74)	39 (76.5)	18 (69.2)	0.493
Presence of associated symptoms	69 (86.3)	47 (88.7)	22 (81.5)	0.377
Number of symptoms, median (IQR)	2 (3–1)	2 (4–2)	2 (3–1)	0.357
1 symptom	20 (29)	12 (25.5)	8 (36.4)	0.514
2 symptoms	22 (31.9)	16 (34)	6 (27.3)	
3 symptoms	12 (17.4)	17 (14.9)	5 (22.7)	
≥4 symptoms	15 (21.7)	12 (25.5)	3 (13.6)	
Sweating	39 (56.5)	31 (66)	8 (36.4)	**0.021**
Nausea	28 (40.6)	17 (36.2)	11 (50)	0.276
Dyspnea	25 (36.2)	16 (34)	9 (40.9)	0.580
Weakness	19 (27.5)	15 (31.9)	4 (18.2)	0.265
Fatigue	17 (24.6)	12 (25.5)	5 (22.7)	0.801
Vomiting	15 (21.7)	10 (21.3)	5 (22.7)	0.892
Dizziness	11 (15.9)	8 (17)	3 (13.6)	1.000
Diarrhea/relaxation of sphincters	5 (7.2)	4 (8.5)	1 (4.5)	1.000
Bouts of feeling hot—chills	3 (4.3)	1 (2.1)	2 (9.1)	0.237
Restlessness	2 (2.9)	2 (4.3)	0 (0)	1.000
Syncope	2 (2.9)	2 (4.3)	0 (0)	1.000
Headache	2 (2.9)	1 (2.1)	1 (4.5)	0.539
Impaired level of consciousness	1 (1.4)	0 (0)	1 (4.5)	0.319
Tingling hands	1 (1.4)	1 (2.1)	0 (0)	1.000

Bold values represent *p* < 0.05 statistically significant.

The results are expressed as median (interquartile range) or *n* (%).

VNRS, verbal numerical rating scale.

In total, 7.5% of the sample were admitted as Killip functional class IV, with circulatory support required in two cases using an intra-aortic balloon pump. Urgent coronary angiography was performed, that is, with activation of the infarction code in 68.8% of cases, preferentially (<24 hours) in 26.3%, and deferred (>24 hours) in 5%. Statistically significant differences were found in the priority of coronary angiography according to the ACS group: urgent in 98.1% of the STEACS versus 11.1% of the NSTEACS, preferential in 1.9% of the STEACS versus 74.1% of the NSTEACS, and deferred only in 14.8% of the NSTEACS group (*p* < 0.001) (Table 4).

**Table 4.** Diagnosis, Therapy, and Complications According to ACS

Variables	Total *N* = 80 (%)	STEACS *N* = 53 (%)	NSTEACS *N* = 27 (%)	*p*-Value
Killip–Kimball classification				
Killip–Kimball I	62 (77.5)	40 (75.5)	22 (81.5)	0.362
Killip–Kimball II	11 (13.8)	9 (17)	2 (7.4)	
Killip–Kimball III	1 (1.3)	0 (0)	1 (3.7)	
Killip–Kimball IV	6 (7.5)	4 (7.5)	2 (7.4)	
Ventricular assist devices in Killip–Kimball IV patients (*n* = 6)				
IABP	2 (33.3)	2 (50)	0 (0)	0.467
Coronarography				
Urgent	55 (68.8)	52 (98.1)	3 (11.1)	**<0.001**
Preferential	21 (26.3)	1 (1.9)	20 (74.1)	
Delayed	4 (5)	0 (0)	4 (14.8)	
ACS etiology				
Obstructive CAD	65 (81.3)	43 (81.1)	22 (81.5)	0.481
MINOCA	14 (17.5)	10 (18.9)	4 (14.8)	
INOCA	1 (1.2)	0 (0)	1 (3.7)	
Therapeutic strategy				
Percutaneous revascularization	58 (72.5)	43 (81.1)	15 (55.6)	**0.012**
Surgical revascularization	6 (7.5)	1 (1.9)	5 (18.5)	
Medical treatment	16 (20)	9 (17)	7 (25.9)	
LVEF classification				
LVEF, median (IQR)	55 (58–45)	55 (57–44)	55 (60–49)	0.087
Reduced (LVEF <40%)	15 (19)	12 (23.1)	3 (11.1)	0.396
Mildly reduced (LVEF 41–49%)	9 (11.4)	5 (9.6)	4 (14.8)	
Preserved (LVEF ≥50%)	55 (69.6)	35 (67.3)	20 (74.1)	
Development of complications of ACS	26 (32.5)	21 (39.6)	5 (18.5)	**0.057**
CA (*n* = 26)	5 (19.2)	5 (23.8)	0 (0)	0.545
Arrhythmias (*n* = 26)	21 (80.8)	16 (76.2)	5 (100)	0.545
Bradyarrhythmias (*n* = 21)	4 (19)	4 (25)	0 (0)	0.532
Tachyarrhythmias (*n* = 21)	17 (81)	12 (75)	5 (100)	
Supraventricular (*n* = 17)	9 (52.9)	6 (50)	3 (60)	1.000
Ventricular (*n* = 17)	8 (47.1)	6 (50)	2 (40)	
Ventricular aneurysm (*n* = 26)	2 (7.7)	2 (9.5)	0 (0)	1.000
Mechanical complications (*n* = 26)	1 (3.8)	1 (4.8)	0 (0)	1.000
More than one complication (*n* = 26)	3 (11.5)	3 (14.3)	0 (0)	1.000

Bold values represent *p* < 0.05 statistically significant.

The results are expressed as median (interquartile range) or *n* (%).

CA, cardiorespiratory arrest; CAD, coronary arterial disease; IABP, intra-aortic balloon pump; INOCA, ischemia with non-obstructive coronary arteries; LVEF, left ventricular ejection fraction; MINOCA, myocardial infarction with non-obstructive coronary arteries.

### Etiology and treatment

The etiology of ACS was secondary to obstructive coronary artery disease in 81.3% of the cases, myocardial infarction with non-obstructive coronary arteries (MINOCA) in 17.5%, and ischemia with non-obstructive coronary arteries in 1.2% (Table 4). The underlying causes of MINOCA included spontaneous coronary artery dissection (64.3%), coronary embolism (21.4%), microvascular angina (7.1%), and the etiology could not be identified (7.1%).

As a therapeutic strategy, percutaneous coronary intervention (PCI) was performed in 72.5% of the cases, referral for cardiovascular surgery in 7.5%, and medical treatment in 20%, with significant differences between the groups (*p* = 0.012). Most STEACS cases were revascularized percutaneously (81.1%), whereas 55.6% of NSTEACS cases were revascularized percutaneously and 18.5% required surgery. During admission, a regulated transthoracic echocardiographic study was performed for prognostic-therapeutic purposes, distributing the cases according to the recommendations of the *European Society of Cardiology*^7^ in three categories based on LVEF: reduced 19%, slightly reduced 11.4%, and preserved 69.6% (Table 4).

### Complications and predictive factors

Complications due to ACS occurred in 32.5% of the population, with differences between groups (STEACS, 39.6% vs. NSTEACS, 18.5%; *p* = 0.057). These were classified as follows: cardiorespiratory arrest (CA), 19.2%; presence of arrhythmias, 80.8%; ventricular aneurysm, 7.7%; and mechanical complications, 3.8% (Table 4). All CAs occurred in the STEACS group, and spontaneous circulation was restored after cardiopulmonary resuscitation maneuvers. Sixty percent of CAs occurred in out-of-hospital settings, and 40% in hospital settings. The initial rhythm was ventricular fibrillation (80%) and polymorphic ventricular tachycardia (20%).

Analysis of arrhythmic complications showed that 19% of the cases presented bradyarrhythmias, in contrast to 81% that showed tachyarrhythmias, 52.9% of which were supraventricular tachyarrhythmias, and 47.1% of which were of ventricular origin, with no differences between the groups (Table 4). It should be noted that all bradyarrhythmias occurred in the STEACS group, more specifically in the STEMIs on the inferior side, with statistical significance compared with the other infarction locations (*p* = 0.031). In addition, 5% of the patients were admitted with subacute myocardial infarction (>24 hours of evolution) and presented a significantly higher prevalence of ventricular aneurysm compared with women with acute myocardial infarction (66.7% vs. 0%; *p* = 0.009).

When analyzing the factors that increased the risk of developing complications derived from ACS, it was observed that women with post-ACS-reduced LVEF had twice the risk of developing complications than those with preserved or slightly reduced LVEF. However, it was found that women who were accompanied during the onset of symptoms had a lower risk of complications than those who were alone (Table 5).

**Table 5.** Estimated Relative Risk for Complications Arising from ACS

Variables	Total *N* = 80 (%)	Presence of complications *N* = 26 (%)	Absence of complications *N* = 54 (%)	*p*-Value	Relative risk	95% CI
Age ≥70 years	41 (51.2)	14 (34.1)	27 (65.9)	0.747	1.110	0.589–2.093
Living alone	22 (27.5)	10 (45.5)	12 (54.5)	0.128	1.648	0.887–3.061
Living in Barcelona (city)	60 (75)	20 (33.3)	40 (66.7)	0.783	1.111	0.520–2.374
Living with dependent persons	19 (23.8)	8 (42.1)	11 (57.9)	0.306	1.427	0.742–2.746
Being with someone else during the acute event	56 (70)	14 (25)	42 (75)	**0.029**	**0.500**	**0.273–0.915**
Perception of severity	32 (40)	12 (37.5)	20 (62.5)	0.436	1.286	0.686–2.409
Imputing the event to cardiac causes	28 (35.9)	10 (35.7)	18 (64.3)	0.479	1.276	0.655–2.484
≥5 CVRFs	42 (52.5)	14 (33.3)	28 (66.7)	0.867	1.056	0.560–1.990
HBP	49 (61.3)	15 (30.6)	34 (69.4)	0.650	0.863	0.457–1.628
DLP	51 (63.7)	14 (27.5)	37 (72.5)	0.201	0.663	0.356–1.236
DM	25 (31.3)	8 (32)	17 (68)	0.949	0.978	0.493–1.941
Post-menopause	69 (87.3)	21 (30.4)	48 (69.6)	0.218	0.609	0.298–1.244
Obesity	23 (28.7)	6 (26.1)	17 (73.9)	0.437	0.743	0.343–1.611
Overweight	30 (37.5)	12 (40)	18 (60)	0.267	1.429	0.765–2.667
Active tobacco use	24 (30)	9 (37.5)	15 (62.5)	0.532	1.235	0.644–2.369
Family history of ischemic cardiomyopathy	33 (41.3)	11 (33.3)	22 (66.7)	0.894	1.044	0.552–1.977
History of ischemic cardiomyopathy	13 (16.3)	1 (7.7)	12 (92.3)	0.051	0.206	0.031–1.390
Mixed anxiety-depressive disorder	28 (35)	10 (35.7)	18 (61.3)	0.652	1.161	0.610–2.207
Perceived stress	29 (36.3)	11 (37.9)	18 (62.1)	0.434	1.290	0.687–2.423
Chest pain	77 (96.3)	24 (31.2)	53 (68.8)	0.245	0.468	0.197–1.112
Killip III/IV	7 (8.8)	4 (57.1)	3 (42.9)	0.206	1.896	0.913–3.936
AMI code activation	55 (68.8)	21 (38.2)	34 (61.8)	0.108	1.909	0.813–4.480
STEMI	53 (66.2)	21 (39.6)	32 (60.4)	0.057	2.140	0.907–5.047
NSTEMI	24 (30)	4 (16.7)	20 (83.8)	0.068	0.424	0.164–1.099
Unstable angina	3 (3.8)	1 (33.3)	2 (66.7)	1.000	1.027	0.201–5.253
Subacute MI	4 (5)	3 (75)	1 (25)	0.098	2.478	1.280–4.799
Obstructive CAD	65 (81.3)	21 (32.3)	44 (67.7)	0.939	0.969	0.437–2.152
MINOCA–INOCA	15 (18.8)	5 (33.3)	10 (66.7)	0.939	1.032	0.465–2.290
Reduced LVEF	15 (19)	8 (53.3)	7 (46.7)	**0.045**	**2.008**	**1.075–3.749**

Bold values represent *p* < 0.05 statistically significant.

The results are expressed as *n* (%).

CI, confidence interval.

A total of 98.8% of the women were discharged from the ACCU, with a median of 2.5 days of admission (interquartile range 4.75–1). Throughout the study period, 5.1% of women were readmitted to the ACCU for different reasons: two were due to decompensated heart failure in <30 days, one to ACS in <30 days, and one to ACS in <6 months. The mortality rate among women included in the study was 1.2%, occurring in one patient in the STEACS group as a consequence of massive cerebral hemorrhage after primary angioplasty. Overall, female mortality rate due to ACS in the unit during the same study period was 2.1% (the patient included and five more patients not included in the study because they were under mechanical invasive ventilation and were not able to do the interview—three after out-of-hospital cardiac arrest and two who were admitted in Killip IV).

## Discussion

Women with ACS admitted to the ACCU are elderly and have a high prevalence of CVRF and chronic comorbidities that require treatment, therapeutic monitoring, and lifestyle changes. This epidemiological profile is similar to that reported by Mauvais-Jarvis et al., in which women who suffered from ischemic heart disease were older than men, and CVRFs such as hypertension, smoking, and diabetes were associated with a higher hazard ratio for myocardial infarction in women.^8^

It is also important to highlight the high percentage of post-menopausal women found in our study, meaning that the risk of CVD in women increases substantially after menopause due to the loss of the protective effect of endogenous estrogens.^4,8^ In this very same sense, premature menopause (<40 years), pregnancy disorders (gestational diabetes, pre-eclampsia, or premature birth), and polycystic ovary syndrome are related to a higher cardiovascular risk.^4^ Likewise, the statistically significant association between active smoking in women and STEACS could be due to the increased risk mentioned above and/or the harmful effects of tobacco, since it produces alterations in the lipid profile, endothelial dysfunction, which is a proinflammatory, prothrombotic, and proarrhythmogenic factor, facilitates atherosclerosis and, in turn, increases the instability of atheromatous plaques. Reyes-Méndez et al. reported that exposure to tobacco smoke (passive smokers) increases the risk of acute myocardial infarction by 25%–31% and produces pathophysiological changes.^9,10^

Smoking is a modifiable CVRF, and smoking cessation is the most effective measure for secondary prevention. On one hand, tobacco dishabituation programs should be started during hospitalization through a combination of behavioral strategies, pharmacotherapy and counseling with a health professional but require individual motivation.^5^ On the other hand, the implementation of tobacco control policies and the dissemination of information about its effects on health are associated with an increase in population-level awareness, encourages quitting the habit, reduces environmental exposure among passive smokers, and reduces cardiovascular morbidity and mortality.^9,10^

It should be noted that, according to our results, most women with a history of ischemic heart disease who experience a new ischemic event do so in the form of NSTEACS. This fact, together with the high cardiovascular comorbidity rate and readmissions recorded throughout the study period, suggests the need to optimize secondary prevention therapies and to design effective interventions aimed at this population group, incorporating psychosocial and gender aspects, promoting empowerment and self-care, and reducing recurrence and, in turn, mortality due to CVD.^4,10^

Although it could also be extrapolated to other societies, the culture of care is directly attributed to women in the European context, and they assume multiple roles that are implicitly associated with gender that can become a barrier to self-care and seeking health care.^4^ The study reflects that almost a quarter of women have people under their care and play the role of primary caregivers.

At the clinical level, according to the *Guideline for the Evaluation and Diagnosis of Chest Pain* of the *American Heart Association*, chest pain is defined as pain, pressure, tightness, or discomfort in the chest, including shoulders, arms, neck, jaw, back, or upper abdomen.^6^ Based on this definition, a high percentage of women in our study reported chest pain during the acute event, being the most common manifestation of ACS in women, although other cofounding symptoms may coexist.

The associated clinical manifestations have mostly been of vegetative nature (sweating, nausea, and vomiting), although there is a significant percentage of symptoms such as dyspnea, weakness, and fatigue that are common among women with ACS and should be considered as anginal equivalents.^6^ These results are in line with the study by Lichtman et al., who concluded that the clinical presentation of acute myocardial infarction in women and men was similar, with chest pain being the predominant symptom in both sexes. They also reported that women presented with a greater number of additional symptoms (more than three), including nausea, indigestion, epigastric pain, dyspnea, and palpitations.^11^

For years, clinical manifestations of ACS that differ from those commonly reported have been labeled “atypical” both in clinical practice and in research studies. This expression is widely used in women and is a confusing descriptor that can induce bias in diagnosis and late access to treatment. Therefore, scientific organizations and various authors have warned about its use.^6,12^

A worrying fact is the low perception of severity, and almost two-thirds of women attribute the symptoms to non-cardiac causes. This phenomenon may be due to multiple factors, such as a gap in knowledge of the symptoms, not identifying or recognizing the individual risk attributable to comorbidities and their influence on cardiovascular health, or because ischemic heart disease remains a male sex stereotype at the population level.^13^

Regardless of the reason, these results are a consequence of a lack of knowledge, have implications for clinical practice, and may cause delays in seeking health care for women with ACS. Therefore, it is necessary to develop gender mainstreaming policies aimed at disseminating and educating the population regarding ACS to promote behavioral and social changes. Furthermore, incorporating gender perspective into medicine involves identifying and combating underestimated diseases.^8^

Women in the study had a higher proportion of STEACS, which is why the infarction code was activated as an established strategy to reduce ischemia time and, in cases with obstructive etiology, access to reperfusion treatment early by PCI, as recommended by the clinical practice guide.^5^

It should be noted that obstructive coronary disease is the fundamental cause of ACS in women; therefore, when symptoms appear, specific diagnostic-therapeutic algorithms should always be activated to reduce ischemia time and mortality. However, other etiologies have been identified, such as MINOCA, which is defined by the presence of ACS symptoms, elevated troponin levels, and non-obstructive coronary arteries. Evidence indicates that MINOCA affects middle-aged people, with a lower prevalence of CVRF, and is common in women and in black, Maori, and Hispanic ethnicities.^14^ Occhipinti et al. reported a lower prevalence than that found in this study, but the clinical profile and the underlying causes of MINOCA, such as spontaneous coronary artery dissection, coronary thromboembolism, and microvascular disease, were collected and described in our study.^14^

Being alone during the onset of symptoms and reduced LVEF were among the factors associated with the appearance of complications in our population. In relation to the latter, it has been found that reduced LVEF post-ACS is a risk factor for the development of complications. Evidence already describes that systolic ventricular dysfunction ≤40% is an independent predictor of ventricular tachycardia and ventricular fibrillation in both STEMI and NSTEMI, and rhythm monitoring for >24 hours is recommended in patients with intermediate to high risk of cardiac arrhythmias.^15^ On the contrary, it has been identified that being accompanied during the acute episode exerts a protective effect against ACS complications. Mirzaei et al. identified women as a risk group because the time interval between the onset of symptoms and the request for health care (decision time) is longer, thus favoring evolving infarcts and worsening the clinical picture.^16^ For this reason, it could be considered that being accompanied would reduce the decision time and encourage the request for assistance at an early stage.

One-third of women presented with complications derived from ACS, highlighting CA, arrhythmias, and conduction disorders. Evidence describes this type of phenomenon in the acute context of ACS, both in the out-of-hospital setting, during coronary revascularization, and in the hospital phase.^15^ Admission to cardiac critical care units is necessary, as they are specialized in acute cardiovascular conditions and the health care staff is trained to manage all aspects and complications of ACS, including arrhythmias, heart failure, mechanical circulatory support, invasive and non-invasive hemodynamic monitoring, respiratory monitoring, mechanical ventilation, and temperature control after CA.^5,17^

One of the limitations identified in this study is selection and survival bias, since the most severe cases that ended up being exitus vitae and those who presented with confusional syndrome after CA could not be included in the study due to the methodological design, which justifies the low rate of mortality, mechanical assistance devices, and invasive mechanical ventilation. In addition, the study was conducted in a single high-complexity center, and the results may not be extrapolated to other clinical environments.

## Conclusions

The clinical and epidemiological profiles of women with ACS admitted to the ACCU were of advanced age, with a high prevalence of CVRF, mainly presenting with STEACS. Chest pain was the predominant symptom with other associated clinical manifestations, and the main etiology of ACS was obstructive coronary disease.

Despite presenting with severe clinical presentations and complications, women have a low perception of severity and attribute symptoms to non-cardiac causes, which may predispose them to a delay in seeking care, especially if they are not accompanied at the time of symptom onset.

Greater awareness of the presence of ACS in women and its complications is needed among the population to improve health outcomes and thus achieve the objectives proposed for 2030.

## Data Availability

The data used in this study are in the possession of the principal investigator.

## Abbreviations Used

ACCU: Acute cardiac care unit
ACS: Acute coronary syndrome
CA: Cardiorespiratory arrest
CVD: Cardiovascular disease
CVRF: Cardiovascular risk factors
LVEF: Left ventricular ejection fraction
MINOCA: Myocardial infarction with non-obstructive coronary arteries
NSTEACS: non-ST segment elevation acute coronary syndrome
NSTEMI: non-ST-elevation myocardial infarction
PCI: Percutaneous coronary intervention
STEACS: ST-segment elevation acute coronary syndrome
STEMI: ST-elevation myocardial infarction
